# Developmental Venous Anomaly Presenting as an Acute Stroke Mimic

**DOI:** 10.7759/cureus.50903

**Published:** 2023-12-21

**Authors:** Arunodaya R Gujjar, Darshan Lal, Sameer Raniga, Amanullah Beg, Abdullah R Al-Asmi

**Affiliations:** 1 Department of Medicine (Neurology), College of Medicine and Health Sciences, Sultan Qaboos University, Muscat, OMN; 2 Department of Medicine (Neurology), Sultan Qaboos University Hospital, Sultan Qaboos University, Muscat, OMN; 3 Department of Radiology and Molecular Imaging, Sultan Qaboos University Hospital, Sultan Qaboos University, Muscat, OMN

**Keywords:** stroke differential diagnosis, stroke mimic, thrombolysis, acute ischemic stroke, developmental venous anomaly

## Abstract

Several mimics of acute ischemic stroke may complicate the decision to administer intravenous thrombolysis. Developmental venous anomalies (DVA) are fairly common variants of normal cerebral vasculature and may occasionally present with acute focal neurologic symptoms. We report a case of DVA presenting with the acute onset of focal neurologic deficits and focal hypodensities on the CT of the brain, resulting in a dilemma regarding whether to administer intravenous tissue-type plasminogen activator (IV tPA) for stroke thrombolysis. Recognition of subtle brain changes on the CT that were inconsistent with an acute ischemic stroke led to further imaging and a definitive diagnosis. Developmental venous anomalies should be considered in the differential diagnosis of acute ischemic stroke.

## Introduction

Acute focal neurologic dysfunction is the hallmark of acute ischemic stroke; however, several other non-vascular conditions may present with acute neurologic symptoms and be misdiagnosed as stroke. Accurate and early recognition of an episode of acute ischemic stroke is now of importance in the context of the routine use of intravenous thrombolysis as an emergent and effective intervention. The occurrence of myriad 'stroke mimics', however, is one of the main reasons that necessitate an initial, careful assessment by a trained neurologist or stroke physician for all such cases before considering emergent thrombolytic therapy [1]. Thrombolysis in non-stroke conditions is contraindicated mainly because it may be futile and expose patients to a risk of hemorrhage. Developmental venous anomalies (DVA) are one of the most common vascular disorders of the brain [2]. We report a case of DVA presenting with the acute onset of focal neurologic deficits, mimicking an acute stroke, resulting in a dilemma regarding whether to administer IV alteplase for stroke thrombolysis.

This case report was presented as an e-poster at the 14th World Stroke Congress held on October 26-29, 2022, in Singapore. 

## Case presentation

A 25-year-old woman presented to our university teaching hospital with a history of sudden left-sided weakness, a mild headache, and altered speech for three hours. She had a cough, rhinorrhea, and transient fever for three days before her current symptoms but had improved. She was not on oral contraceptive medication. There was no history supporting a seizure. Examination revealed a pulse of 112/min and a blood pressure of 102/56 mmHg. The cardiac examination was unremarkable. Neurologically, she was alert and oriented, with mild dysarthria. She had a partial left upper motor neuron facial palsy and left upper limb weakness of grade 4/5 on the Medical Research Council (MRC) scale, with left pronator drift and left-sided hypoesthesia. Plantar responses were flexor. The National Institutes of Health (NIH) Stroke Scale score was six. Blood glucose, complete blood counts, and routine biochemistry were normal. An emergent real-time reverse transcriptase-polymerase chain reaction (RT-PCR) test for COVID-19 virus infection was negative.

**Figure 1:**
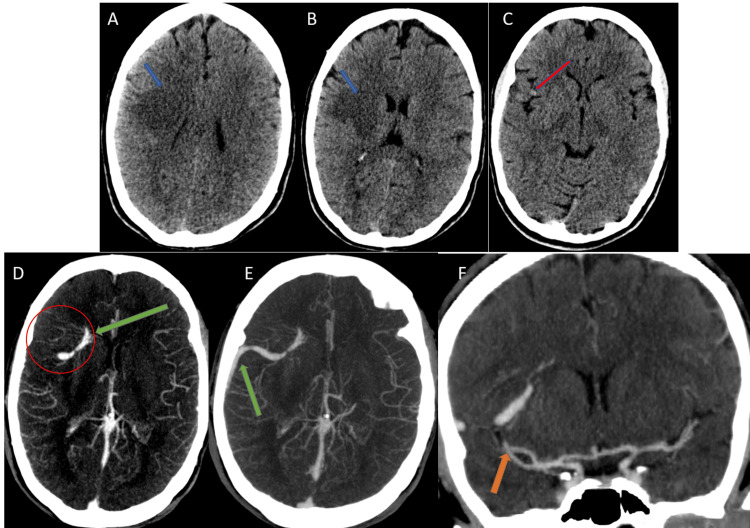
A CT scan of the brain and CT angiogram of a 25-year-old female patient who presented with acute left faciobrachial weakness of three hours duration. A plain CT scan of the brain (A, B, and C) showed an ill-defined area of hypoattenuation seen in the right frontal deep white matter (A, B) with effacement of the sulci. A hyperattenuating vascular structure was seen (C) in the right Sylvain fissure (arrows). A CT angiography performed five hours after the onset of symptoms (D, E, and F) showed a curvilinear-enhancing aberrant vascular structure extending from the right frontal horn to the Sylvain fissure and draining into the right transverse sinus (arrow in D and E). A few abnormal medullary veins draining into the beginning of this vessel were also noted (D, E, and F). Normal M1 and M2 segments of the right MCA were noted (F) (arrow).

The possibility of stroke in a young person involving the right internal carotid artery territory was considered. An emergent plain CT brain study three hours after symptom onset showed a right frontal hypodensity involving the right middle cerebral artery territory (Figures 1A-1B).

However, thrombolysis with recombinant tissue plasminogen activator (rtPA) was withheld on suspicion of a subtle hyperdensity noted in the right Sylvian fissure region (Figure 1C) as well as focal brain edema predominantly in the white matter but sparing the cortex.

A contrast brain CT and CT angiogram performed soon after showed patent carotid and vertebral arteries, as well as bilateral middle cerebral arteries. It also revealed an abnormally dilated vascular channel beginning at the tip of the right frontal horn and extending initially laterally along the Sylvian fissure and then posteriorly along the brain surface to drain into the lateral part of the right transverse sinus (Figures 1D-1F). The presence of a DVA was suspected, and IV alteplase administration was withheld thereafter.

**Figure 2:**
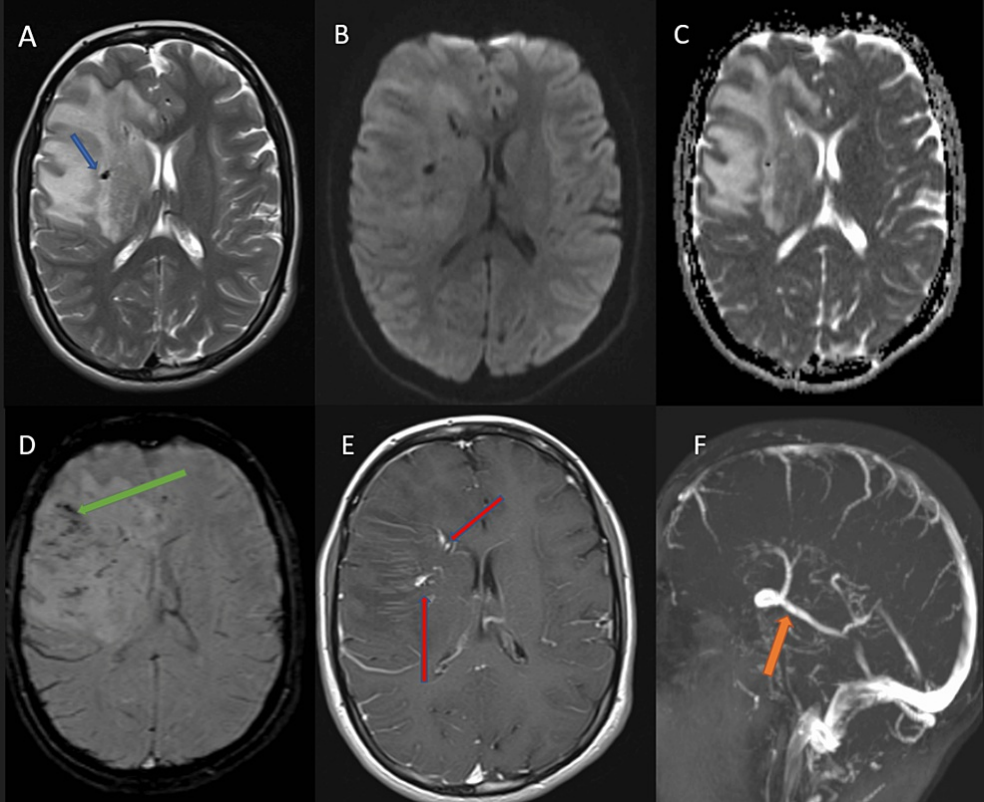
Brain MRI of the patient performed 24 hours after symptom onset The T2 weighted axial image (A) showed extensive vasogenic edema in the right frontal lobe with a mass effect. A vascular flow void (arrow) was noted within the edematous region. Diffusion-weighted imaging (DWI) (B) and apparent diffusion coefficient (ADC) axial (C) images showed no diffusion restriction. The susceptibility-weighted image (D) and post-gadolinium T1 axial image (E) showed abnormal veins forming a radial array of “caput medusae” appearance (red arrow), converging to the beginning of the dilated collector vein. The collector vein was patent in the MR venogram (F), coursing back along the brain surface, and draining into the right transverse sinus.

An MRI and MR angiographic study performed about 24 hours after the stroke onset showed extensive right frontal T2 hyperintensities (Figures 2A-2C), suggesting evolving focal brain edema likely in the region drained by the DVA.

The susceptibility-weighted (SWI) study showed dilated medullary veins within the frontal lobe hyperintense region, converging towards the tip of the frontal horn and draining into the collector vein with a course, as demonstrated on the contrast CT (Figures 2D-2F). No evidence of thrombosis of the cortical veins or dural venous sinuses was observed. A digital subtraction angiographic study performed three days after symptom onset confirmed the diagnosis of DVA with no evidence of venous thrombosis. Echocardiography and the Holter study showed no cardiac disorders as potential sources of embolism.

Possible diagnoses considered at this stage were a complicated DVA with contralateral hemiparesis, cerebral venous thrombosis, or 'posterior reversible encephalopathy' syndrome. However, extensive brain imaging showed no convincing evidence of venous thrombosis; she was normotensive and had no seizures. An electroencephalogram (EEG) was not performed in the context of no observed seizure before presentation or at the hospital (and partly due to restrictions imposed by COVID-19 hospital protocols). The patient was initiated on IV mannitol in view of the evidence of focal brain edema with contralateral hemiparesis. A review on day three of admission revealed persistent weakness and some progression of edema on MRI; at this stage, IV dexamethasone was initiated empirically. She improved significantly over the next five days, and she was discharged.

**Figure 3:**
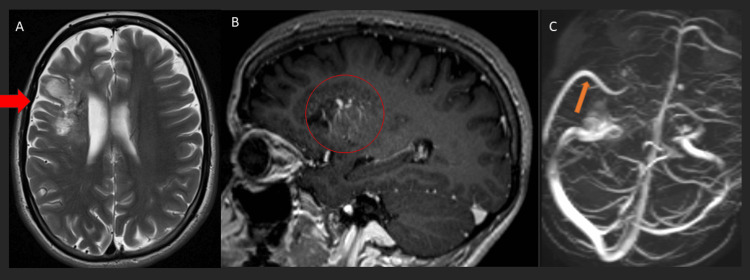
Brain MRI of the patient in the context of a developmental venous anomaly at the one-year follow-up. The T2 weighted axial image (A) showed significant regression of the vasogenic edema in the right frontal lobe with encephalomalacia and gliosis. However, the post-gadolinium T1 axial image (B) and MR venogram 3D image (C) showed persistent medullary draining veins and a large collector vein draining into the right transverse sinus, representing the developmental venous anomaly.

At the clinical follow-up four weeks later, she was asymptomatic. After a detailed consideration of the likely mechanism leading to her brain changes and symptoms, the possibility of acute thrombosis of the venous channels of the DVA draining the left frontal lobe was considered, though no overt thrombus could be demonstrated on imaging. She was initiated on anticoagulation with oral rivaroxaban, which was continued for one year. Apart from a brief episode of a left focal motor seizure lasting a few minutes, (three months after the presentation of initial symptoms) which was treated with levetiracetam, she remained well. A repeat MRI of the brain one year later (Figure 3) showed focal right frontal atrophy with gliosis and persistence of the entire DVA.

## Discussion

This is an unusual case of a young woman with an acute focal neurologic deficit (left faciobrachial weakness) and a right frontal hypodensity, initially diagnosed as an evolving acute ischemic stroke in the left middle cerebral artery territory. An incidental upper respiratory infection was proven not to be due to the COVID-19 virus. The immediate dilemma was whether to offer thrombolysis with IV alteplase for this patient. The initial clue to the diagnosis was the presence of focal brain edema in a non-arterial territory distribution. Further CT and MRI imaging studies led to a conclusive diagnosis of DVA and to deferring thrombolytic therapy.

Developmental venous anomalies are considered to be extreme variations of normal cerebral veins. They typically consist of converging, dilated, medullary veins that drain radially into a transcerebral collector vein that finally drains either into the normal superficial or deep venous sinuses [2-4]. Estimates of DVA prevalence range from roughly 5% to 10% [5, 6]. While most DVAs are asymptomatic, a few rarely present with neurologic symptoms [4]. Pereira et al. [7] reported 69 patients with symptomatic DVAs, all studied by MRI, and proposed possible underlying mechanisms leading to symptomatic DVAs which were mechanical obstruction in 20%; increased blood flow in 28%; decreased outflow (e.g., due to thrombosis) in 38%; increased venous pressure in 5%; and an unknown mechanism in 9%. Our patient likely developed symptoms due to increased venous pressure, possibly due to altered hemodynamics or unrecognized downstream venous thrombosis. The acute evolution of symptoms with focal non-hemorrhagic cerebral edema in the territory of dilated venous channels was considered to be due to possible thrombosis in the distal venous channels, which resolved over a few days spontaneously, rather than arteriovenous shunting, which is usually a gradual and enduring process. Such DVAs, even with no overt hemorrhage or infarction, are reported to be associated with headaches, transient focal neurologic deficits, and seizures, as in our patient [4]. Developmental venous anomalies may thus present with varied clinical symptoms due to different mechanisms of brain injury. Conservative and symptomatic management is recommended in most instances. Progressive or worsening conditions attributable definitively to DVAs may be considered for micro- or radio-surgery, with an emphasis on preserving normal venous channels [4].

The purpose of this case report is to highlight the possibility of DVA presenting as a stroke mimic. A recent seizure, hypoglycemia, cerebral venous thrombosis, cerebral contusion, glioma, metastasis, and MELAS (short for mitochondrial encephalomyopathy, lactic acidosis, and stroke-like episodes) are all examples of 'stroke mimics' [1]. A presentation resembling acute ischemic stroke or transient ischemic attacks due to DVA has been rarely reported in the literature. However, no such reports were associated with the consideration of possible thrombolysis [4, 8, 9]. The consequences of thrombolysis with IV alteplase in the context of a DVA are unknown. To our knowledge, this is the first report of a DVA presenting as an acute stroke mimic, leading to the dilemma of considering thrombolytic therapy. Developmental venous anomalies should be considered among stroke mimics in patients with acute ischemic stroke who are being considered for thrombolysis or endovascular interventions.

## Conclusions

Developmental venous anomalies are relatively common variants of normal cerebral vasculature. They may occasionally manifest with clinical symptoms mimicking acute stroke due to complications such as thrombosis, venous congestion, or parenchymal hemorrhage. Here we present a case report where focal neurologic deficits and focal brain hypodensity due to DVA led to a dilemma of considering IV thrombolysis for suspected ischemic stroke. Recognition of subtle brain changes on the CT that were inconsistent with an acute ischemic stroke led to a definitive diagnosis of DVA. Developmental venous anomalies with complications should be considered in the differential diagnosis of acute ischemic stroke.
